# Method for visualizing detailed profiles of synchrotron X-ray beams using diamond-thin films and silicon drift detectors

**DOI:** 10.1107/S1600577525002838

**Published:** 2025-04-22

**Authors:** Togo Kudo, Shinji Suzuki, Mutsumi Sano, Toshiro Itoga, Hiroyasu Masunaga, Shunji Goto, Sunao Takahashi

**Affiliations:** a Japan Synchrotron Radiation Research Institute Sayo-cho 1-1-1 Sayo-gun Hyogo679-5198 Japan; b RIKEN SPring-8 Center (RSC), Sayo-cho 1-1-1, Sayo-gun, Hyogo679-5148, Japan; Paul Scherrer Institut, Switzerland

**Keywords:** X-ray beam profile monitors, silicon drift detectors, undulators, diffraction-limited storage rings, X-ray beam position monitors, front-end slits

## Abstract

Contamination from nearby bending magnet radiation hinders precise and accurate determination of the true beam center of undulator radiation. To solve this problem, a semi-nondestructive method was developed to visualize the detailed profile of a synchrotron radiation beam by using a thin diamond film as a scatterer. As the beam passed through the diamond film, scattered X-rays were imaged using a pinhole camera and measured with a two-dimensional silicon drift detector (SDD) scan. With this configuration, the beam center was accurately determined by visualizing the radiation pattern distribution for each energy level of a pink X-ray beam within an aperture size of 1.5 mm × 1.5 mm, shaped by a front-end slit (FES) positioned upstream of the monochromator. Additionally, by scanning the FES in two dimensions with a reduced aperture of 0.4 mm × 0.4 mm, energy-resolved images were successfully obtained using the SDD at a fixed position. These images revealed the profile of undulator radiation over a broad area (with an aperture extending up to 4 mm) in a pre-slit positioned upstream of the FES, demonstrating good alignment with *SPECTRA* calculations. This method effectively eliminates contamination from nearby bending magnet radiation, a significant issue in previous approaches, enabling a direct and highly accurate determination of the true beam center.

## Abstract 2

The presented measurement using a silicon drift detector allows us to observe the photon beam center of undulator radiation without the influence of bending magnet radiation.

## 1. Introduction

Traditionally, the determination of the beam center of synchrotron radiation emitted from undulators has been challenging owing to contamination from bending magnets positioned upstream and downstream of the undulator. To address this, some synchrotron facilities have introduced corrections to X-ray beam position monitor (XBPM) measurements for each undulator gap value (Shu *et al.*, 1998 ▸) and have modified magnet arrangements to minimize the contamination of undulator radiation by bending magnet radiation (Decker & Singh, 1999 ▸). Despite advancements in beam monitoring technology that reduce the impact of bending magnet radiation, eliminating this interference remains a challenge (Yang *et al.*, 2013 ▸). Consequently, a new method is needed to determine accurately the X-ray beam center.

Recent studies have highlighted the importance of energy-resolved observations for accurate measurements of the true X-ray beam center (Takahashi *et al.*, 2016 ▸; Goto *et al.*, 2007 ▸). Building on the accumulated knowledge from these studies, we have successfully visualized the radiation profile of an undulator beam using a pinhole camera equipped with an energy-resolved two-dimensional detector (Kudo *et al.*, 2020 ▸, 2022 ▸). This approach confirmed that the undulator radiation profile aligns well with the predicted profile. Note that this profile has been previously approximated only via simulations using *SPECTRA* (Tanaka, 2021 ▸). Conventional XBPM methods estimate the beam center indirectly from available information based on the radiation surrounding the center using four-blade sensors. Consequently, radiation contamination from bending magnets has considerably affected the measured values (Aoyagi *et al.*, 2004 ▸). The proposed method employs energy resolution to filter out bending magnet radiation, making it easier to determine accurately the true beam center.

Another advantage of this method is that it can be installed in an optics hutch downstream of the front-end slit (FES). Compared with the inner parts of the accelerator tunnel, the optics hutch has lower radiation levels and better access, making it easier to maintain the equipment. The downstream location of the FES narrows the aperture and reduces the heat load on the diamond thin film.

Notably, the energy resolution of the 2D detector has a considerable impact on the performance of the proposed synchrotron beam profile measurement method. Our previous study used the SOPHIAS 2D detector (Hatsui & Graafsma, 2015 ▸) with an energy resolution full width at half-maximum (FWHM) of 2 keV to successfully capture radiation pattern images. However, the resolution of these images was sub­optimal owing to the insufficient energy resolution (Kudo *et al.*, 2022 ▸). Thus, calculating the beam centroid without a large FES aperture remains challenging (Kudo *et al.*, 2022 ▸, 2020 ▸). However, a large FES aperture hinders inline beam diagnostics during operation, as the FES aperture must be small to prevent unnecessary heat loading on the downstream optics system.

In this study, the undulator beam was visualized in detail through scanning measurements using a silicon drift detector (SDD), which offered superior energy resolution. Herein, we present two methods for energy-resolved visualization of undulator beams using an SDD. Using these methods, we found the beam center downstream of the FES aperture. These measurements can be adapted to achieve the initial alignment of the beamline.

## 2. Experimental setting

The X-ray beam was visualized using two methods involving the placement and movement of an SDD and an FES. In the first method, the SDD, with an attached pinhole, was scanned using a pulse motor and provided high-spatial resolution for beam monitoring. In the second method, the FES was scanned, while the SDD remained fixed, enabling energy-resolved visualization across a broader spatial range of undulator radiation.

Experiments were conducted in the optics hutch of the frontier soft-material beamline at SPring-8 BL03XU (Masunaga *et al.*, 2011 ▸). The light source was a standard SPring-8 in-vacuum undulator (magnet period length of 3.2 cm and total length of 4.5 m) (Kitamura, 1998 ▸). The FES was located ∼30 m downstream from the source (Oura *et al.*, 1998 ▸). The SDD, with an energy resolution of Δ*E* = 140 eV (FWHM), was calibrated using ^109^Cd (*K*α: 5.9 keV) and ^55^Fe (*K*α: 22.1 keV) radioactive sources to ensure linearity within the energy range of the undulator’s primary radiation.

### 2.1. Measurement of the X-ray beam center of the undulator beam with high-spatial resolution based on SDD scanning

**Figure 1:**
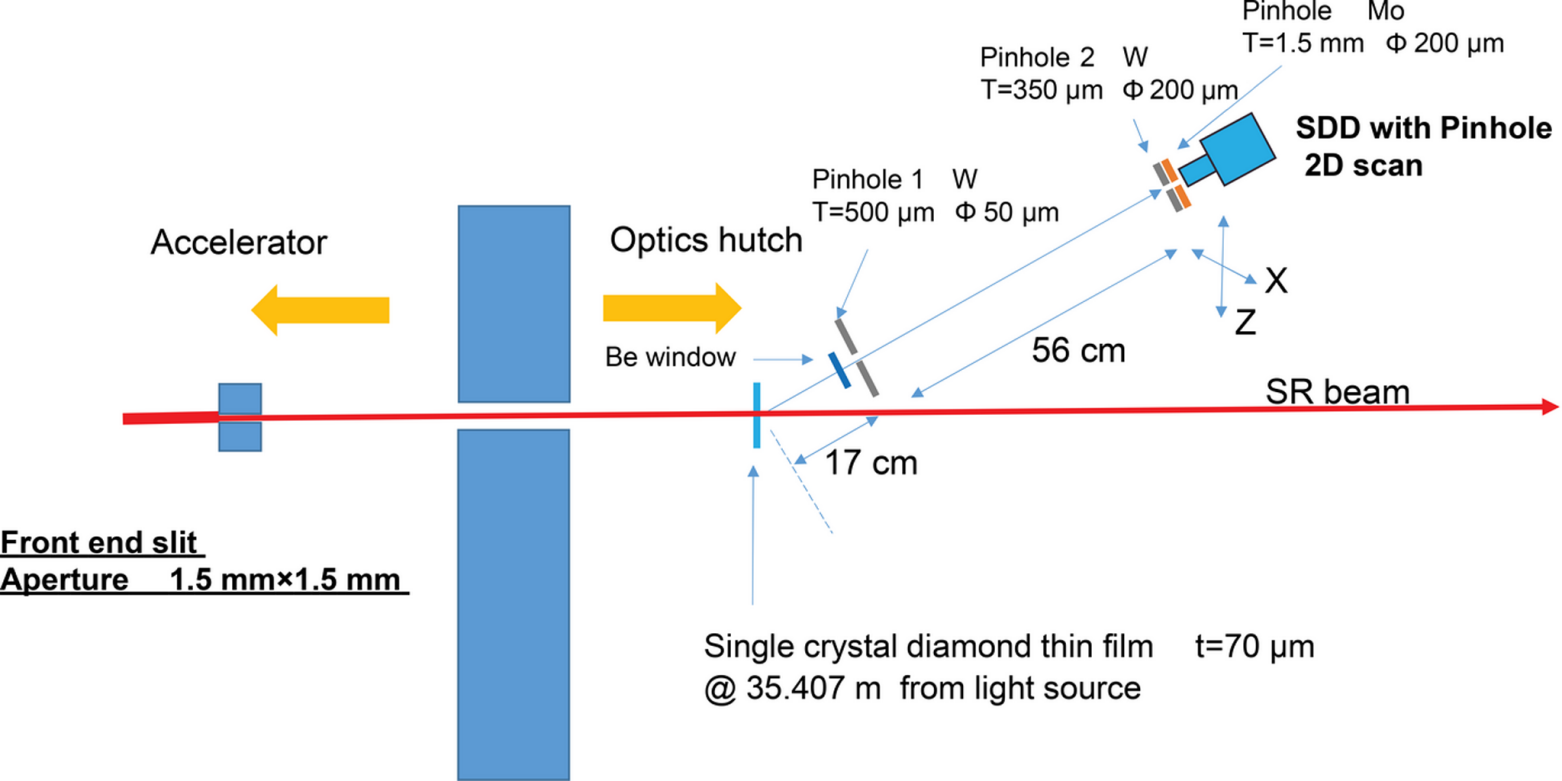
Schematic drawing of the BL03XU optics hutch setup for a high-spatial-resolution X-ray beam monitor. All the components are in vacuum, except for the SDD and pinholes 1 and 2.

Fig. 1 ▸ shows the setup within the optics hutch. A pink synchrotron radiation beam was directed through a thin single-crystal diamond film (thickness: 70 µm) (the diamond film was purchased from EDP, https://www.d-edp.jp/en/) housed in a vacuum chamber at 35.407 m from the light source. Forward-scattered X-rays were extracted through a viewport with a Be window (thickness: 120 µm) at an upward angle of 30°. Pinhole 1, made from 0.5 mm-thick tungsten with a 50 µm diameter hole, was placed near the Be window. The hole had a through-hole thickness of 150 µm and a 60° countersunk facing the opposite side of the vacuum chamber. The distance from the diamond to pinhole 1 was 17 cm. The spectrum of scattered radiation passing through pinhole 1 was measured using the SDD, positioned 56 cm away. The SDD used was the XSDD50-01 model from Techno AP (sensor thickness: 400 µm; effective area: 50 mm^2^), equipped with a digital signal processor (DSP) from Techno AP (APU101X). The DSP settings were flat top = 200 ns and rise time = 50 ns. These DSP parameters were set to ensure a high-count rate to obtain an energy resolution sufficiently smaller than the spectral width of the undulator. A second pinhole, pinhole 2 (diameter: 200 µm), was attached to the effective area of the SDD to determine the position of incident photons. This pinhole, comprising tungsten (thickness: 350 µm) backed with 1.5 mm molybdenum, improved the spectrum by absorbing tungsten fluorescence near the SDD, as confirmed by *PHITS* 3.10 simulations (*PHITS*, https://phits.jaea.go.jp/indexj.html). The SDD and pinhole 2 moved together, forming a scanning pinhole camera with a magnification of 3.3×. A vacuum path was maintained between the Be window and the SDD.

The pulse motor driving the *X* and *Z* stages of the SDD moved in 200 µm increments per measurement point in both horizontal and vertical directions, with a fixed FES aperture of 1.5 mm × 1.5 mm. The 2D scan pitch was equal to the size of pinhole 2. Thus, this method constructed an image similar to that obtained with a 2D detector, with pixel sizes equivalent to the size of pinhole 2. In our configuration, a point source at the diamond film formed a spot (diameter: ∼200 µm) at the SDD, which was 4.3 times larger than the diameter of pinhole 1. The diameter of pinhole 2 (200 µm), equal to the scan pitch, was chosen to maximize photon capture while maintaining the spatial resolution of the pinhole camera. In this system, pinhole 1 (diameter: 50 µm), which was positioned at the exit of scattered X-rays in the vacuum chamber, established the spatial resolution limit.

The storage ring current was set to 100 mA and the undulator gap was fixed at 26 mm (17.3 keV at the first harmonic). A program was developed using *LabVIEW* (developed by D-Studio Co., https://d-studio21.com/) to synchronize the motor drive and data acquisition of the SDD scan. The integration time for each point was set to 5 s.

### 2.2. Wide-field measurement using FES scans

**Figure 2:**
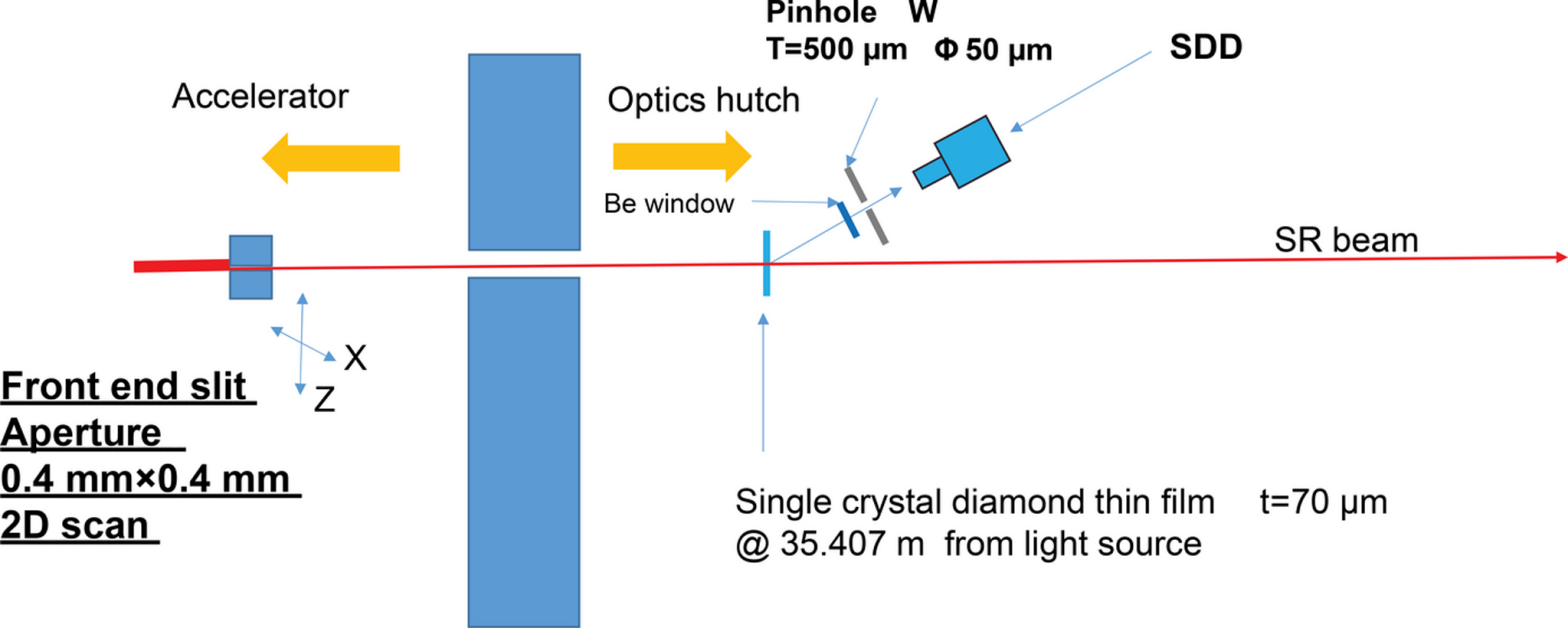
Schematic drawing of the BL03XU optics hutch setup for a large field-of-view X-ray beam monitor. All components are in vacuum, except for the SDD and pinhole.

Using a fixed SDD configuration and FES scans, the undulator radiation was visualized across a broad field of view. As shown in Fig. 2 ▸, the scattered radiation passing through the pinhole was measured using a stationary SDD positioned nearby. The pinhole and DSP settings used were the same as those described in Section 2.1 for pinhole 1. The aperture of the FES was set to 0.4 mm × 0.4 mm. The FES was scanned in 2D across 5 mm horizontal and vertical ranges, with a pitch of 0.1 mm, while spectra were acquired with the SDD. The scanning of the FES, a beamline component controlled by the *Message and Database Oriented Control Architecture* (*MADOCA*) (Tanaka *et al.*, 1995 ▸), was executed by sending *MADOCA* commands to the beamline workstation. The *LabVIEW* program was used to synchronize the FES scan and SDD data acquisition. The X-ray exiting the pinhole moved by <1 mm at the imaging plane during the scanning process. The SDD with a diameter of 8 mm then captured all the X-rays passing through the pinhole. The storage ring current was set to 100 mA and the undulator gap was fixed at 14.8 mm (10 keV at the first harmonic). The integration time for each point was set to 1 s.

## 3. Results

### 3.1. Measurement of high-spatial resolution images

**Figure 3:**
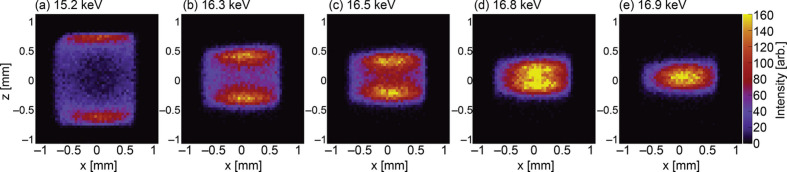
Scanned images of the high-spatial resolution beam monitor.

Fig. 3 ▸ presents the measured results obtained using the setup in Fig. 1 ▸. A total of 2000 spectra were analyzed to calculate the energy-resolved intensity and compiled into a 2D map. Each image represents an energy-resolved image of ten bins corresponding to 67 eV. The total measurement time for this experiment was 4 h. Fig. 3 ▸ shows that the beam splits vertically at low energy [Fig. 3 ▸(*a*)] and gradually converges toward the center as the energy increases and approaches the primary radiation peak [Figs. 3 ▸(*b*)–3 ▸(*e*)]. Additionally, the radiation in Fig. 3 ▸(*a*) allows us to recognize the shape of the FES aperture, which is 1.5 mm × 1.5 mm. Comparing the profiles in Figs. 3 ▸(*a*) and 3 ▸(*e*) shows that energy-resolved imaging yields a considerably smaller vertical beam size. In the vertical direction, the X-ray beam position can be measured accurately even downstream of the FES.

**Figure 4:**
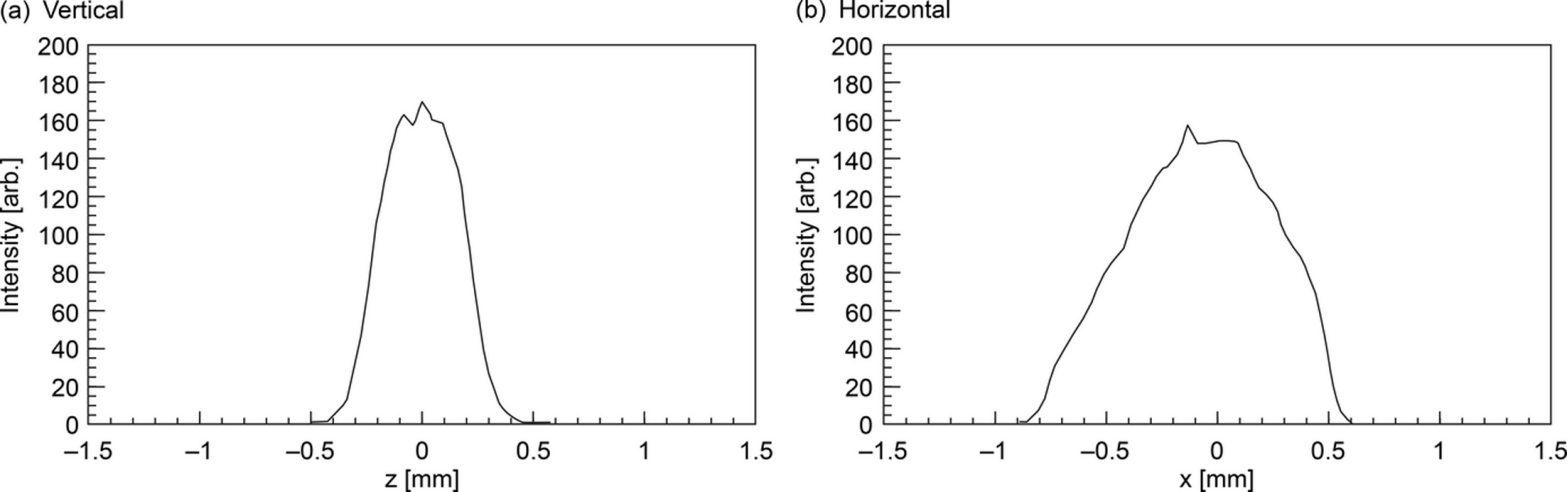
Beam profile at primary radiation peak (*E* = 16.9 keV).

Fig. 4 ▸ shows the beam profile at *E* = 16.9 keV, derived from the data presented in Fig. 3 ▸. In the vertical direction, the beam size (FWHM ≃ 500 µm) is considerably smaller than the FES aperture of 1.5 mm. The vertical beam center remained measurable, even when the FES aperture was narrowed to 1 mm × 1 mm. Conversely, in the horizontal direction, the aperture slightly masks the beam tail because the original beam size was larger.

### 3.2. Measurements of large spatial area images

**Figure 5:**
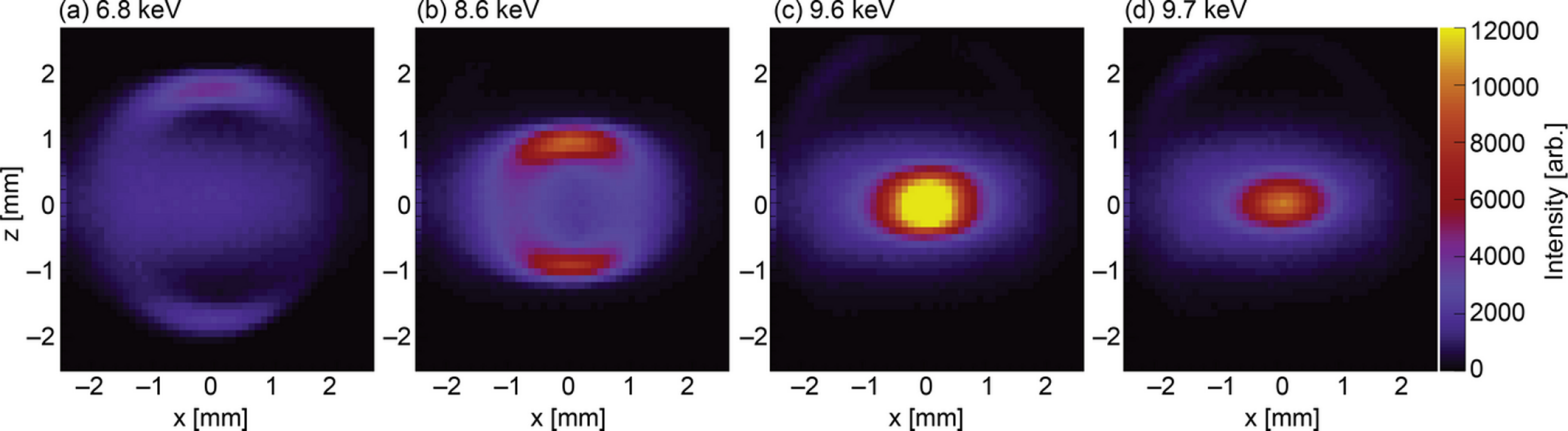
Beam images of the first harmonics.

**Figure 6:**
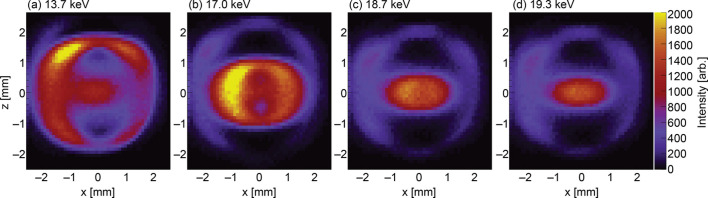
Beam images of the second harmonics.

**Figure 7:**
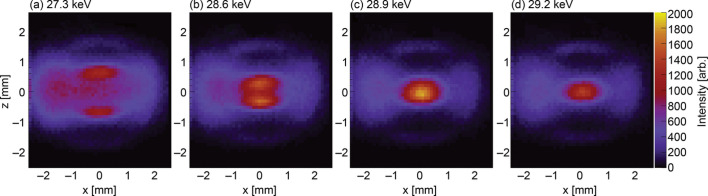
Beam images of the third harmonics.

Figs. 5 ▸–7 ▸ ▸ show the measurement results obtained using the setup shown in Fig. 2 ▸. In total, 2500 spectra were analyzed to calculate the energy-resolved intensity, which was then compiled into a 2D map. The total measurement time for this experiment was 6 h. Each image represents an energy-resolved image of ten bins corresponding to 80 eV. The profiles up to the third harmonic are shown. The beam images at the first harmonics of the undulator spectrum initially split vertically at low energies and converged toward the beam center as energy increased (Fig. 5 ▸). Regarding the second-order harmonics, the beam concentrated at the beam center and formed a ring shape (Fig. 6 ▸). Regarding the third-order harmonics, the beam converged again from the top and bottom (Fig. 7 ▸). These images show the full beam profile after passing through the pre-slit, a component with an aperture of 4 mm located before the FES (Takahashi *et al.*, 1998 ▸), and their energy distributions are in good agreement with the *SPECTRA* calculations.

A relatively large FES aperture size of 0.4 mm × 0.4 mm, which is larger than the spatial resolution of 50 µm (Section 2.1), was used in the measurements. To obtain more detailed images, we reduced the FES aperture to 0.2 mm × 0.2 mm; this resulted in an asymmetric horizontal profile and led to considerable image degradation. Figs. 5 ▸–7 ▸ ▸ show that the beam profile is asymmetrical in the horizontal direction. This means that even an aperture size of 0.4 mm × 0.4 mm is still insufficient. The asymmetry in the beam profiles becomes more pronounced as the FES aperture is reduced. Conversely, this asymmetry can be alleviated by increasing the aperture size. However, in this case, the image resolution degrades. One cause of this asymmetry is that the FES is a long object along the beam (*L* ≃ 2 m). The FES used an inclined incidence design to withstand the intense heat load of the undulator radiation. Accordingly, the kick of beam divergence on the left and right sides is difficult to equalize.

**Figure 8:**
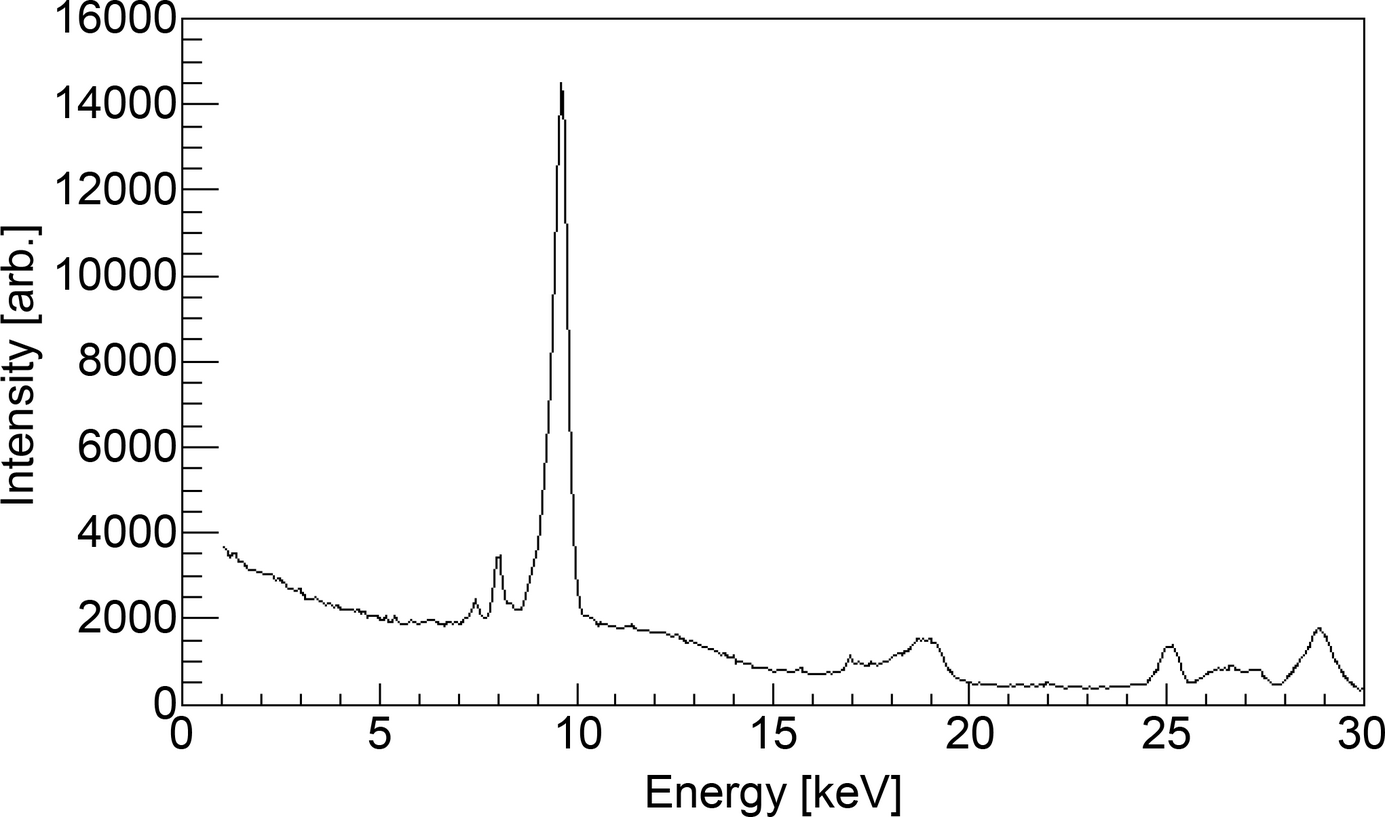
Spectrum obtained at an undulator gap setting of 14.8 mm.

Fig. 8 ▸ shows the spectrum at the beam center, extracted using a 0.4 mm × 0.4 mm FES opening under the same conditions as those used to generate Figs. 5 ▸–7 ▸ ▸. The first harmonic of the undulator spectrum radiation peak was observed at 9.7 keV (*SPECTRA* calculation yielded 10 keV). The second and third harmonic peaks were observed at 19.3 keV and 29 keV, respectively. Additionally, the peak at 8 keV was ascribed to the fluorescence of Cu, which was used in the brazing sections of the cooling pipes and thin diamond film clamps.

**Figure 9:**
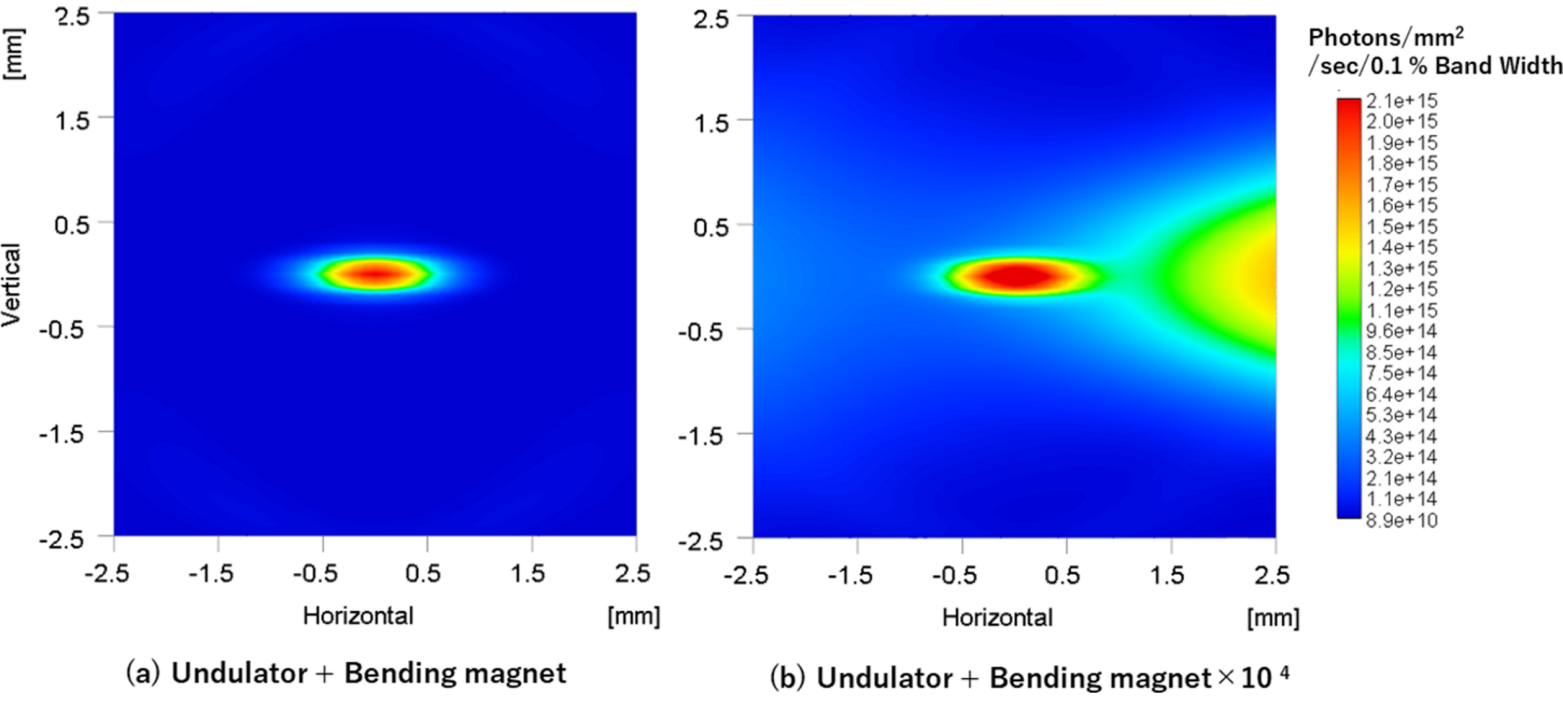
Simulation of the radiation pattern with gap = 14.8 mm at 10 keV using *SPECTRA*.

Fig. 9 ▸(*a*) shows the radiation pattern simulated using *SPECTRA* for an undulator with a gap of 14.8 mm, along with the radiation from the upstream and downstream bending magnets of the undulator. The calculation results are presented after monochromatization at 10 keV. Note that when the energy is resolved, the radiation from the bending magnets is significantly suppressed and is almost indiscernible on the same scale. Fig. 9 ▸(*b*) presents the same diagram; however, the radiation from the bending magnets is magnified by a factor of 10000. This amplification enables the recognition of the radiation from the bending magnets.

## 4. Discussion

This study used an SDD to examine energy-resolved beam profiles using two distinct methods, independent of measurement time constraints. By using the SDD with high-energy resolution (Δ*E* = 140 eV), we obtained detailed beam shapes compared with previous energy-resolved images acquired using a 2D detector (Δ*E* = 2 keV) (Kudo *et al.*, 2022 ▸). In both methods, the images did not observe bending magnet radiations on either side of the undulator beam center. *SPECTRA* simulations confirmed that with high energy resolution, photon flux from bending magnet radiation contamination near the beam center was reduced by approximately four orders of magnitude compared with that of the undulator radiation. The energy-resolved beam observation technology demonstrated here eliminated the effects of bending magnet radiation on the X-ray beam center measurement.

To observe the undulator radiation pattern of high-order harmonics, measuring the profile with narrow gaps is preferable. However, we performed measurements at different gap values in the SDD and FES scans considering the heat load on the optics system. In this study, the FES aperture was broadened in the SDD scan to obtain the beam profile with the broadest possible field of view; this resulted in an increased heat load on the downstream optics system. Consequently, the heat load can only be reduced by widening the undulator gap. Conversely, in the FES scan, the FES aperture had to be relatively narrow to achieve good spatial resolution; this reduced the heat load on the downstream optics, making it possible to perform measurements with a narrower gap. These are limitations due to the cooling performance of the double-crystal monochromator installed downstream of the diamond film. Improvements in the cooling system allowed the SDD scans to measure narrower-gap undulator beam profiles than those measured using FES scans.

A limitation of the FES scan is that the spatial resolution is restricted by the FES aperture size of 0.4 mm. In terms of spatial resolution, the SDD scans were superior to the FES scans. However, to observe an undulator beam with a narrow gap in an SDD scan, the downstream optics must be sufficiently cooled. A limitation of both of these methods is their long measurement times. Therefore, calculating the beam position using this information at high speed, as well as incorporating it into the feedback that stabilizes the light source, is difficult. Currently, this measurement can be adapted to the initial alignment of the beamline. However, using a 2D detector with excellent energy resolution (Hatsui & Graafsma, 2015 ▸; Grimes *et al.*, 2023 ▸), faster measurements are possible.

Multi-element SDDs (Utica *et al.*, 2021 ▸) are preferred over 2D detectors when a simple and fast calculation of the centroid of an energetically resolved beam is required. Although multi-element SDDs have larger pixels than those of typical image detectors, they provide valuable information for observing the beam centroid of the first harmonic of the undulator spectrum at the peak energy within practical measurement times. In this case, the measurement time of 4 h in the SDD scan can be reduced considerably to several seconds by a multi-element SDD.

## 5. Conclusions

We visualized the synchrotron beam profile with an energy resolution of Δ*E* = 140 eV using a pinhole camera arrangement optimized for precise beam-center measurements and an FES scan arrangement suitable for wide-area radiation visualization.

The first method determined the spatial resolution limitation using a pinhole size of 50 µm.

In the second method, a 2D scan of the FES aperture (0.4 mm × 0.4 mm) enabled the visualization of energy-resolved radiation profiles across a broad field of 5 mm × 5 mm.

The measurements were adapted for the initial alignment of the beamline.
